# Erosive pustular dermatosis–like scalp reaction following cranial radiotherapy in a patient with EGFR-mutant NSCLC treated with amivantamab

**DOI:** 10.18632/oncoscience.649

**Published:** 2026-03-11

**Authors:** Vasiliki Nikolaou, Antonis Tsimpidakis, Ioannis-Alexios Koumprentziotis, Evdoxia Panou, Theodoros Tegos, Alexander Stratigos

**Affiliations:** ^1^First Dermatology Department, “A.Sygros” Hospital for Skin Diseases, National and Kapodistrian University of Athens Medical School, Athens, Greece; ^2^Medical Oncology Department, Evangelismos General Hospital, Athens, Greece; ^3^Medical Oncology Department, Hygeia Hospital, Athens, Greece

**Keywords:** amivantamab, lung cancer, cutaneous toxicity, erosive pustular dermatosis, radiotherapy

## Abstract

Amivantamab, a bispecific monoclonal antibody directed against the epidermal growth factor receptor (EGFR) and mesenchymal–epithelial transition factor (MET), has demonstrated significant clinical activity and is increasingly incorporated into frontline regimens for EGFR-mutant non–small cell lung cancer (NSCLC). Its increasing clinical use has, nevertheless, been paralleled by recognition of distinctive cutaneous adverse events, most commonly acneiform eruptions, paronychia, and xerosis among others. We present the first case of an erosive pustular dermatosis (EPD)-like reaction limited to a previously irradiated field in a patient receiving amivantamab for EGFR-mutant NSCLC. This case draws attention to the importance of recognizing EPD-like scalp ulcerations as a potential adverse event in patients receiving amivantamab, events that have not been recognized in clinical trials, particularly in the setting of recent or concurrent radiotherapy.

## INTRODUCTION

As targeted therapies increasingly represent a cornerstone of management for epidermal growth factor receptor (EGFR)-mutant non–small cell lung cancer (NSCLC), new patterns of toxicity continuously emerge that require multidisciplinary diagnosis and management. Amivantamab, a bispecific monoclonal antibody directed against the EGFR and mesenchymal–epithelial transition factor (MET), has demonstrated significant clinical activity and is increasingly incorporated into frontline regimens. Its increasing clinical use has, nevertheless, been paralleled by recognition of distinctive cutaneous adverse events, most commonly acneiform eruptions, paronychia, and xerosis [1, 2].

In addition to these expected EGFR-related toxicities, more unusual ulcerative scalp lesions have been reported to develop in up to 15% of patients receiving amivantamab [3]. Clinically, these lesions manifest as crusted erosions and superficial ulcers overlying erythematous or pustular plaques and are notable for their acute onset, aggressive clinical course, and frequent secondary infection, often causing pain and impaired quality of life. They share very similar clinical and histopathologic features with erosive pustular dermatosis (EPD)—a chronic neutrophilic dermatosis that occurs predominantly in elderly men with severe actinic damage, typically affecting chronically sun-damaged areas such as the scalp [4]. We report, to our knowledge, the first case of an EPD-like reaction limited to a previously irradiated field in a patient receiving amivantamab for EGFR-mutant NSCLC.

## CASE REPORT

A 39-year-old woman was diagnosed in May 2023 with metastatic lung adenocarcinoma with EGFR L858R mutation, PD-L1 negative, and ALK negative. Baseline imaging revealed brain, bone (Th3), and pulmonary metastases, with no abdominal involvement. She received first-line osimertinib together with palliative radiotherapy (Th2–Th5). After approximately 11 months of treatment, she had disease progression with new lesions in the mediastinum and liver and was subsequently changed to amivantamab with carboplatin and pemetrexed according to the Phase III MARIPOSA-2 protocol [5]. She continued to receive maintenance amivantamab and pemetrexed following four cycles of induction, with radiologic stabilization of disease.

**Figure 1:**
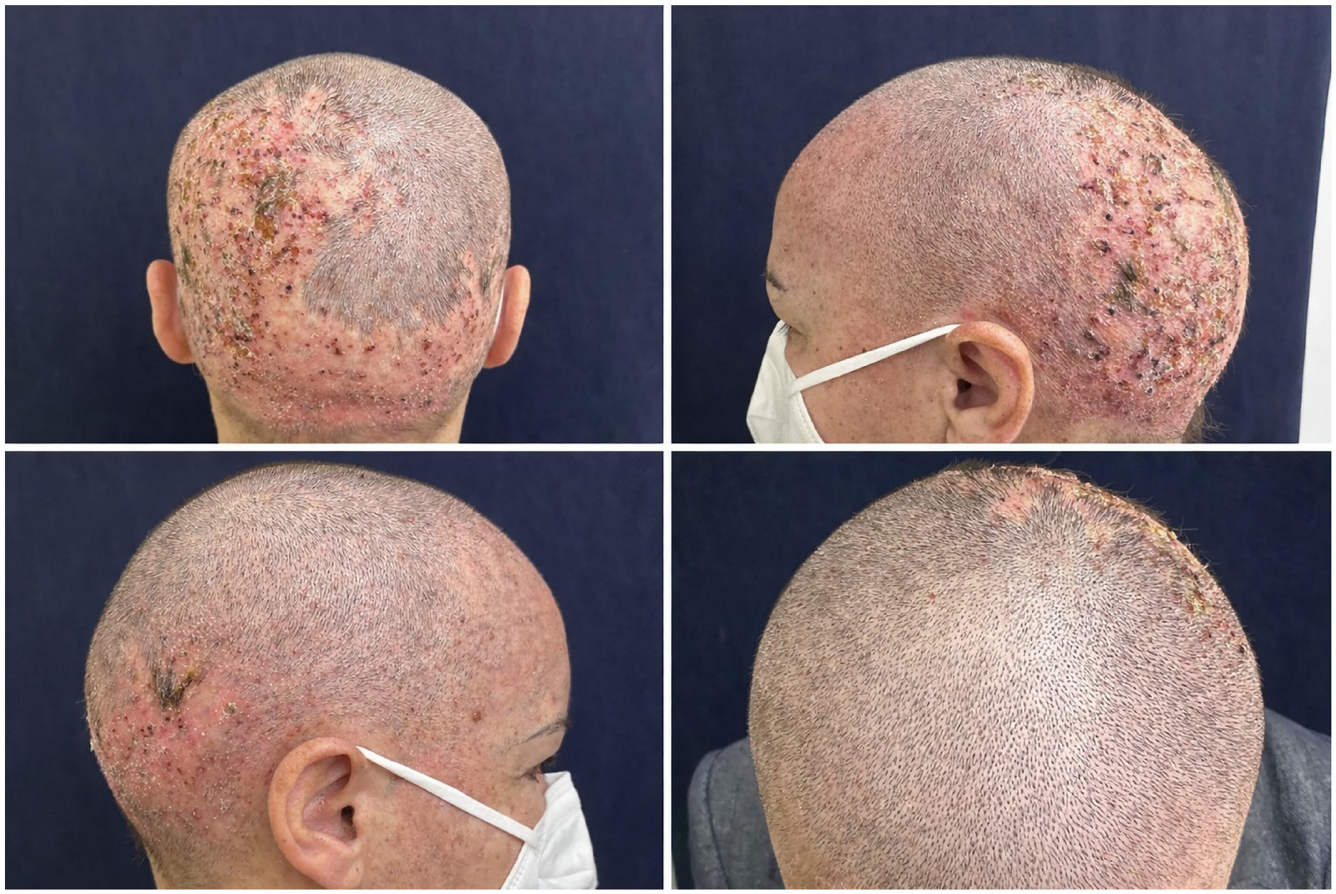
Painful, crusted erosive plaques confined to the irradiated scalp region in patient under amivantamb treatment.

Eighteen months into systemic therapy, she presented to our oncodermatology clinic with a grade-2 acneiform eruption over the face and trunk. She had received tetracycline therapy for approximately one year, achieving partial improvement; this was replaced by oral dapsone (100 mg/day), resulting in complete clearance of the lesions. Four months later, with the eruption in remission, she developed central nervous system oligoprogression and underwent cranial radiotherapy while staying on amivantamab treatment. Despite ongoing remission of her acneiform rash and while still on dapsone treatment, she subsequently developed painful, crusted erosive plaques confined to the irradiated scalp region, clinically consistent with erosive pustular dermatosis of the scalp (EPDS) (Figure 1). No other cutaneous sites were affected. Bacterial cultures were negative, and the lesions gradually responded to high-potency topical corticosteroids applied twice daily, topical corticosteroids and antiseptic dressings without any interruptions in amivantamab dosing. The patient did not discontinue dapsone and did not experience a recurrence of the acneiform rash at last follow-up.

## DISCUSSION

This case draws attention to the importance of recognizing EPD-like scalp ulcerations as a potential adverse event in patients receiving amivantamab, particularly in the setting of recent or concurrent radiotherapy. Misdiagnosis of similar rashes as infectious, necrotic, or post-radiation dermatitis may delay appropriate management and may lead to unjustified interruption of oncologic therapy.

Interestingly, in our case, the patient had been on amivantamab for almost two years but experienced this rare cutaneous adverse event only after undergoing cranial radiotherapy. Moreover, the patient was receiving dapsone therapy for the previously developed acneiform rash, a treatment option that has been successfully used in cases of EPD-like scalp ulcerations due to amivantamab [6] and still developed ulcerative lesions.

The pathophysiology of these reactions likely reflects the complex interplay between local epidermal injury, radiation-induced inflammation, and impaired epithelial repair secondary to EGFR/MET inhibition. Both the EGFR and MET signaling pathways are important in keratinocyte migration and wound healing; dual inhibition may act synergistically with radiation-induced injury, predisposing to ulcer formation. Recognition of this potential radiotherapy–amivantamab interaction is of clinical interest to thoracic oncologists, since amivantamab is being increasingly paired or sequenced with local brain radiation in EGFR-mutant disease.

Because of this possible interaction, proactive scalp management may be warranted in patients scheduled for cranial irradiation while undergoing treatment with amivantamab. Regarding prevention, measures involve minimizing mechanical irritation, moisturizing the scalp and initiating prophylactic topical corticosteroids or anti-inflammatory agents within the radiation field.

If EPD-like reactions occur, based on the limited available literature, treatment options may include combinations of adequate topical care, topical and oral corticosteroids, and oral tetracyclines with the goal to avoid the discontinuation of amivantamab [6, 7].

In all cases, early dermatologic consultation is required for proper diagnosis and management of ulcerative scalp reactions.

## CONCLUSIONS

In summary, this report presents a case of a novel EPD-like scalp eruption limited to a previous radiotherapy field in a patient treated with amivantamab. Recognition of this new toxicity by oncologists, dermatologists, and radiation oncologists is crucial, as early diagnosis and local treatment can allow for ongoing life-extending targeted therapy with reduced morbidity. Additional research is needed to understand the mechanisms of these reactions and to determine preventative measures for patients undergoing concurrent or sequential radiotherapy and amivantamab.
